# Herpes Simplex Virus Pneumonia Mimicking Legionella Pneumonia in an Elderly Patient With Heart and Liver Failure

**DOI:** 10.7759/cureus.21938

**Published:** 2022-02-05

**Authors:** Miki Yamashita, Ryuichi Ohta, Naoto Mouri, Sho Takizawa, Chiaki Sano

**Affiliations:** 1 Family Medicine, Shimane University Medical School, Izumo, JPN; 2 Community Care, Unnan City Hospital, Unnan, JPN; 3 Obstetrics and Gynecology, Shimane University Medical School, Izumo, JPN; 4 Community Medicine Management, Shimane University Faculty of Medicine, Izumo, JPN

**Keywords:** rural hospital, older patients, liver cirrhosis, heart failure, legionella, pneumonia, herpes simplex virus

## Abstract

The diagnosis of viral pneumonia is often difficult because of its varied presentations. Regarding the serological diagnosis of viral infections, it is difficult to perform a viral DNA test in general medical facilities, especially in rural settings. Among viral pneumonia cases, herpes simplex virus (HSV) pneumonia can occur in immunocompromised hosts. However, when the clinical course of HSV pneumonia is acute, and the features of pneumonia are not distinct, the diagnosis can be challenging. We report the case of a 69-year-old man who visited the hospital with complaints of dyspnea and cough for two days. Although the patient had no fever and the urine was negative for Legionella antigen, we suspected Legionella pneumonia based on the clinical course, Gram stain of sputum, and CT findings. After undergoing treatment with antibiotics, his condition worsened, with dyspnea and an increase in the demand for oxygen at 5 L. The patient also had complications related to the heart and liver. The sputum culture was negative, and the HSV serum test revealed that HSV IgM level was elevated to 1.16 (reference value: ≤0.80) and IgG level at admission and at follow-up 21 days later was elevated to 28.1 and 60.0 respectively (reference value: ≤2.0); accordingly, the patient was diagnosed with HSV pneumonia. In cases of pneumonia with atypical courses, the coexistence of multiple diseases, and immunosuppression, HSV pneumonia should be included in the differential diagnosis. In rural settings, viral pneumonia should be investigated using antibodies against viruses due to the limited availability of other medical resources. When a patient is judged to be immunosuppressed in the treatment of pneumonia of unknown cause, it is important to consider the possibility of HSV infection and take prompt action. Furthermore, it is essential to consider the possibility of multi-organ failure secondary to HSV infection, which requires systemic management.

## Introduction

The diagnosis of viral pneumonia is often difficult because of its varying presentations. In general, causative pneumonia viruses consist of colonized viruses such as adenovirus or rhinovirus, and most cases are mild [1]. Viral pneumonia is rarely differentiated from upper respiratory tract inflammation [1]. The clinical course of viral pneumonia is slowly progressive, similar to other viral diseases, and may follow a subacute to chronic course depending on the degree of inflammation [2]. It is necessary to distinguish it from atypical pneumonia, such as pneumonia caused by mycoplasma [3]. The diagnostic imaging of viral pneumonia is also unclear, and in recent years, coronavirus disease 2019 (COVID-19) pneumonia imaging has gained significant attention [4,5]. They are now sometimes featured as images of viral pneumonia.

Furthermore, regarding the serological diagnosis of viral infections, it is difficult to perform a viral DNA test in general medical facilities, especially in rural settings [2]. The antibody titer against each virus is often measured and performed as a provisional diagnosis [1]. Moreover, the diagnosis of viral pneumonia may be difficult when the causative agent is a rare virus, such as the herpes simplex virus (HSV) [6]. Among viral pneumonia cases, HSV pneumonia can occur in immunocompromised hosts. For the diagnosis of HSV pneumonia, it is important to evaluate the immune status of patients in whom pneumonia is triggered [7]. Various diseases affect patients’ immunity, such as cancers and other chronic organ failures [8]. In some cases, the presentation of viral pneumonia can be mild, with a clinical course similar to that of the common cold in response to supportive treatments [1].

However, when the clinical course of HSV pneumonia is acute, and the features of pneumonia are not distinct, the diagnosis can be challenging. Furthermore, the conditions of immunity may modify the presentation of pneumonia. In this report, we present a case of HSV pneumonia in a patient with heart failure and hepatic failure. It was difficult to distinguish the condition from Legionella pneumonia at the initial diagnosis stage. This report highlights the process of diagnosing HSV pneumonia in patients with multiple diseases and discusses effective treatment methods.

## Case presentation

A 69-year-old man presented to the hospital with complaints of dyspnea and cough for two days. The patient’s symptoms had been progressive, prompting him to visit our hospital. He had visited a hot spring four days before the presentation. However, he had not interacted with anyone there. He had not been exposed to patients suspected of having severe acute respiratory syndrome coronavirus 2 (SARS-CoV-2) infection. His medical history included hypertension, type 2 diabetes, dyslipidemia, alcoholic cirrhosis, and heart failure, and his last medical follow-up had been a year ago.

**Figure 1:**
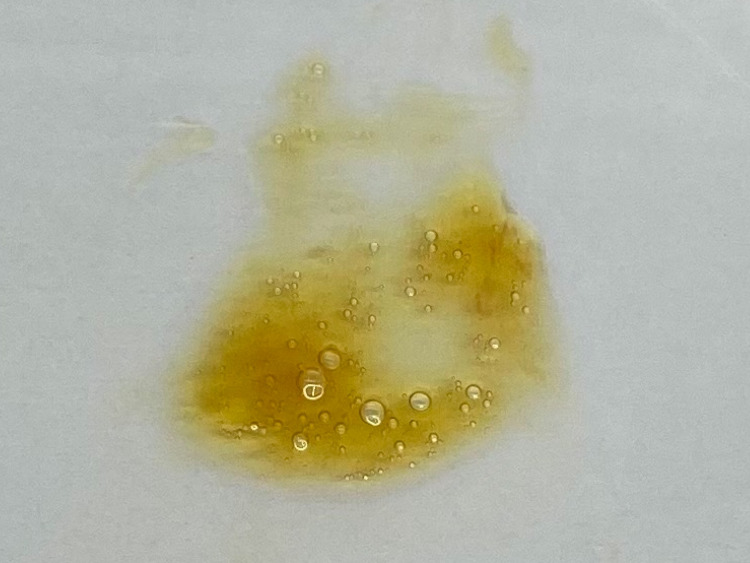
The finding of orange-colored sputum

**Figure 2:**
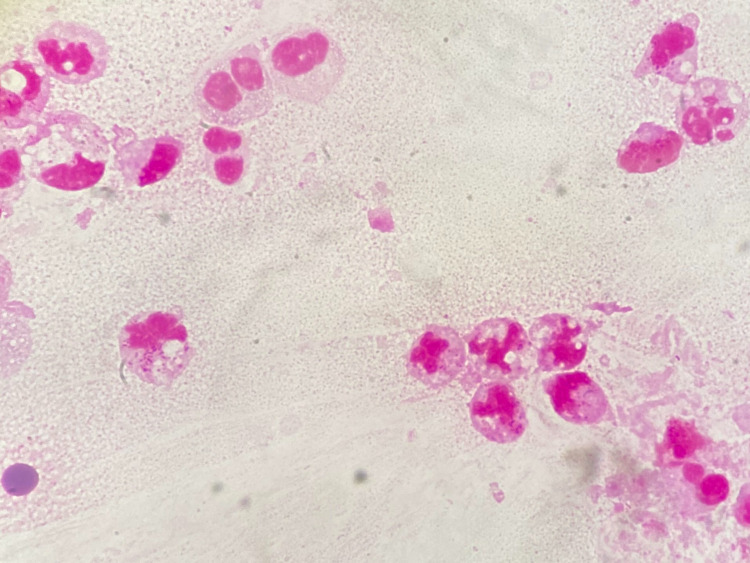
The Gram stain of the sputum showing leukocytes without bacteria

At the time of admission, the patient’s vital signs were as follows - body temperature: 36.2 °C; blood pressure: 142/86 mmHg; pulse rate: 123 beats/minute; SpO_2_: 93% on room air; and respiratory rate: 30 breaths/minute. Physical examination revealed peripheral warmth and crackles in the bilateral lung fields. He had no obvious heart murmur or leg edema. The patient’s sputum was orange-colored (Figure 1). Gram-staining of the sputum showed leukocytes without bacteria (Figure 2).

The patient's blood tests revealed high leukocyte levels and high inflammatory findings, elevated hepatic enzymes, hyponatremia, and increased creatine kinase (Table 1).

**Table 1:** Initial laboratory data of the patient PT-INR: prothrombin time-international normalized ratio; APTT: activated partial thromboplastin time; eGFR: estimated glomerular filtration rate; CK: creatine kinase; CRP: C-reactive protein; Ig: immunoglobulin; HCV: hepatitis C virus; SARS-CoV-2: severe acute respiratory syndrome coronavirus 2; HBs: hepatitis B surface; EBV VCA: Epstein-Barr virus capsid antigen; EBNA: Epstein-Barr virus nuclear antigen

Marker	Level	Reference values
White blood cells	15.30	3.5–9.1 × 10^3^/μL
Neutrophils	70.1	44.0–72.0%
Lymphocytes	11.4	18.0–59.0%
Monocytes	17.9	0.0–12.0%
Eosinophils	0.4	0.0–10.0%
Basophils	0.2	0.0–3.0%
Red blood cells	3.64	3.76–5.50 × 10^6^/μL
Reticulocytes	5.8	/μL (%)
Hemoglobin	13.3	11.3–15.2 g/dL
Hematocrit	38.5	33.4–44.9%
Mean corpuscular volume	106.6	79.0–100.0 fL
Platelets	12.4	13.0–36.9 × 10^4^/μL
PT-INR	1.53	
APTT	36	25–40 s
Erythrocyte sedimentation rate	35	2–10 mm/h
Total protein	7.4	6.5–8.3 g/dL
Albumin	2.7	3.8–5.3 g/dL
Total bilirubin	2.8	0.2–1.2 mg/dL
Direct bilirubin	1.5	0–0.4 mg/dL
Aspartate aminotransferase	95	8–38 IU/L
Alanine aminotransferase	41	4–43 IU/L
Alkaline phosphatase	124	106–322 U/L
γ-glutamyl transpeptidase	472	<48 IU/L
Lactate dehydrogenase	479	121–245 U/L
Blood urea nitrogen	29	8–20 mg/dL
Creatinine	1.81	0.40–1.10 mg/dL
eGFR	31.4	>60.0 mL/min/L
Serum Na	114	135–150 mEq/L
Serum K	4.0	3.5–5.3 mEq/L
Serum Cl	80	98–110 mEq/L
Serum Ca	8.2	3.5–5.3 mg/dL
Serum P	3.2	0.2–1.2 mg/dL
Serum Mg	1.9	1.8–2.3 mg/dL
Ferritin	229.1	14.4–303.7 ng/mL
CK	175	56–244 U/L
CRP	4.71	<0.30 mg/dL
IgG	1,104	870–1,700 mg/dL
IgM	161	35–220 mg/dL
IgA	832	110–410 mg/dL
HBs antigen	0.00	0.00–0.04 IU/mL
HCV antibody	0.09	0.00–0.99 S/CO
SARS-CoV-2 antigen	-	
SARS-CoV-2 PCR	-	
EBV VCA IgG	7.9	<0.5
EBV VCA IgM	0.7	<0.5
EBV EBNA IgG	3.2	<0.5

**Figure 3:**
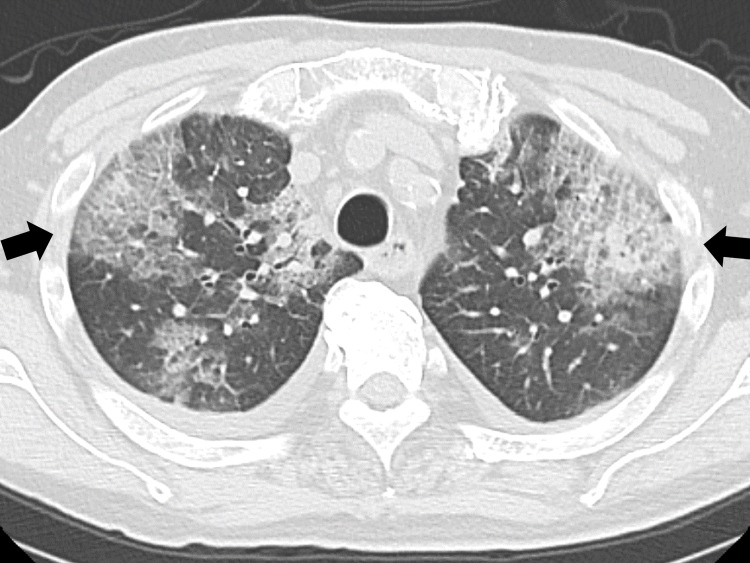
Chest CT showing bilateral pleural effusion and pan-lobular infiltration shadows (arrows) CT: computed tomography

His chest CT showed bilateral pleural effusion and pan-lobular infiltration shadows (Figure 3).

Although the patient had no fever and the urine sample was negative for Legionella antigen, we suspected community pneumonia, including Legionella pneumonia, based on the clinical course, sputum findings, and CT findings. We initiated treatment with intravenous ceftriaxone (2 g) and oral azithromycin.

**Figure 4:**
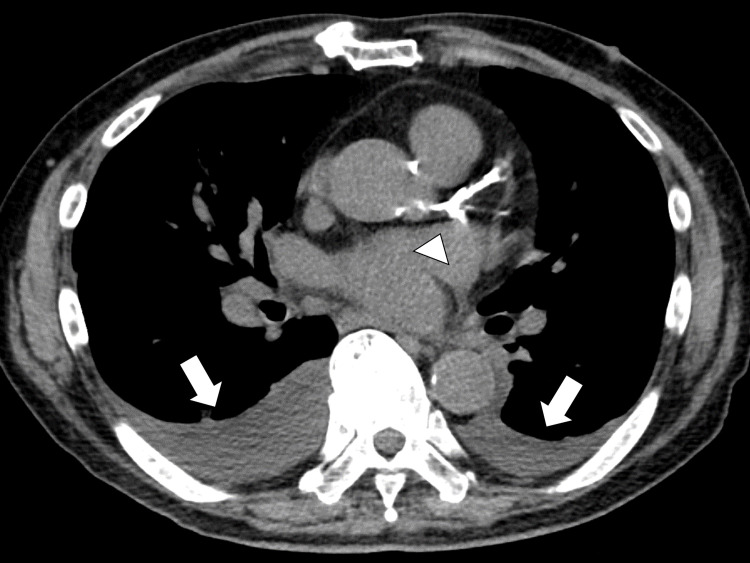
Chest CT showing bilateral pleural effusions (white arrow) and calcification of coronary arteries (white arrowhead) CT: computed tomography

On the day of admission, the patient’s blood pressure was maintained, and bilateral leg edema was observed. Pleural effusions were observed on chest CT (Figure 4).

The patient was diagnosed with exacerbation of heart failure, and furosemide 20 mg was administered. The EKG showed ST elevation on VR and ST depression on V4-5, I, and II inductions at the time of admission. The next day, pleural effusion was drained due to respiratory failure The result was a leaky pleural effusion. The initial blood test showed a high brain natriuretic peptide level of 1,172.7 ng/mL and troponin I level of 1.429 ng/mL. Transthoracic echocardiography revealed a decrease in the left ventricle contractility (ejection fraction: 0.21), especially in the circumflex region.

Considering the possibility of myocardial ischemia, the patient was referred to the Department of Cardiovascular Medicine to evaluate the coronary arteries. In the cardiology department, acute heart failure due to relative myocardial ischemia was diagnosed based on increased oxygen demand triggered by pneumonia as per the chest image and disease course. A previous chest CT had shown coronary artery calcification (Figure 4); coronary angiography was not performed, and continuous administration of heparin and nicorandil was initiated.

**Figure 5:**
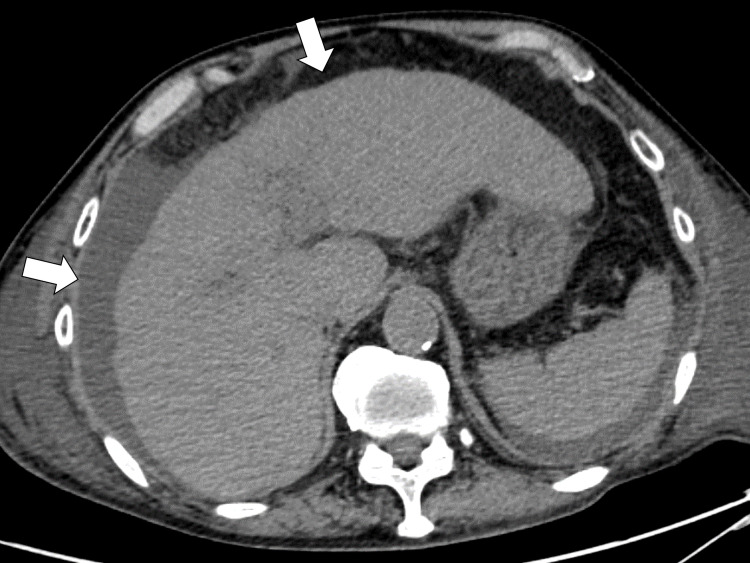
Abdominal CT showing liver cirrhosis with ascites accumulation on the surface of the liver (arrows) CT: computed tomography

The patient had a history of hospitalization for alcoholic liver disease and had dropped out from regular follow-ups approximately one year before this visit. At the time of admission, liver function abnormalities were high, accompanied by hypoalbuminemia and hyponatremia. He was diagnosed with decompensated cirrhosis with Child-Pugh classification C based on the non-alcoholic fatty liver disease score of 3.14 (>0.665) and fibrosis-4 (FIB-4) index score of 5.25 (>2.67). Three days after the admission, the patient complained of exacerbation of abdominal distension. Five days after the admission, abdominal CT showed liver cirrhosis with ascites accumulation on the surface of the liver (Figure 5).

The puncture was performed with an aspiration of 2,600 mL of effluent and pale-yellow ascites. The serum ascites albumin gap was 2.0, showing that the cause of the ascites was an exacerbation of right heart failure, an increase in portal vein pressure due to the progression of liver cirrhosis, and a decrease in oncotic pressure due to low albumin levels (approximately 2.0).

The antibiotic was changed from azithromycin to levofloxacin from the second day after admission because of the exacerbation of dyspnea and increased oxygen demand at 5 L, which impacted his swallowing ability. On the 10th day of hospitalization, levofloxacin and oxygen administration were terminated. The sputum culture was found to be negative, and the HSV serum test revealed that the HSV IgM level was elevated to 1.16 (reference value: ≤0.80), and the IgG level was elevated to 28.1 (reference value: ≤2.0). Therefore, HSV pneumonia was suspected. As the patient’s symptoms did not exacerbate and the risk of exacerbation of liver cirrhosis and renal failure was considered, we spared the usage of antiviral drugs for the exacerbation; 14 days later, HSV IgG level was elevated to >60 (reference value: ≤2.0).

Subsequently, the patient showed no exacerbation of symptoms. Acute heart failure and liver failure were alleviated simultaneously. Continued nutritional therapy and rehabilitation improved his condition, and he was discharged home independently.

## Discussion

In this study, we encountered a case of slow-onset progressive dyspnea that led to the diagnosis of HSV pneumonia while the patient concurrently underwent empirical treatment. The medical history of patients with HSV pneumonia can be complex; therefore, the disease and the subsequent treatments may cause complications in the heart and liver. It is important to continue monitoring the systemic symptoms of HSV pneumonia. In addition, as the clinical course of HSV pneumonia remains unclear, it can be difficult to differentiate it from atypical pneumonia. It is necessary to consider the clinical course and multifaceted findings, such as sputum Gram staining, during diagnosis.

In cases of pneumonia with atypical courses, the coexistence of multiple diseases, and immunosuppression, HSV pneumonia should be included in the differential diagnosis. In our patient, we suspected atypical pneumonia rather than typical pneumonia based on CT imaging findings and slowly progressive respiratory symptoms. However, no clear diagnostic picture was obtained from clinical findings [2,9]. In the clinical course of our patient, the lack of response to broad-spectrum antimicrobial agents, widespread diffuse infiltrative shadows and slit glass shadows on chest CT, poor improvement of slit glass shadows, and elevated lymphocytes and monocytes on blood sampling suggested the possibility of viral pneumonia.

In rural contexts, viral pneumonia should be investigated using antibodies against viruses because of the limited availability of other medical resources. In general, HSV pneumonia can be difficult to diagnose, and polymerase chain reaction (PCR) testing of sputum can be used for the diagnosis [1]. However, HSV may reside in the upper respiratory tract, and a positive PCR test may not necessarily indicate infection [6,10]. Serum IgM assay indicates the likelihood of HSV pneumonia [10], and serum immunoglobulin tests can be performed in rural settings. Rural areas have many older patients vulnerable to viral infection, and older people are nowadays choosing to access rural medical care rather than travel to urban areas due to the COVID-19 pandemic [11]. Immunoglobulin tests should be performed in rural contexts in older patients with pneumonia even if they do not reveal clear etiologies.

Furthermore, the clinical course of the disease is the key in diagnosing acute pneumonia of unknown origin. In bacterial pneumonia, symptoms often resolve relatively quickly with the administration of antibiotics [12]. Conversely, atypical pneumonia and viral pneumonia may show an acute progressive course and a slowly progressive course, which may offer a clue to the diagnosis [1]. Older patients may not have typical symptoms and may be reluctant to use medical care [13-16]. Furthermore, COVID-19 has affected rural older people’s help-seeking behaviors [17]. Therefore, without precise investigation, pneumonia in older patients can be missed. If the sputum Gram stain shows a low bacterial count relative to the white blood cell count, the possibility of viral pneumonia should be considered.

HSV infection may become severe in patients with various illnesses, which may lead to exacerbation of coexisting diseases of the liver, kidney, and heart [18,19]. In our case, the patient had liver cirrhosis and chronic renal failure, suggesting that he was immunocompromised. His respiratory condition gradually worsened, and at the same time, his myocardial and liver markers were elevated. Considering the possibility that the patient was infected with HSV, the elevated levels of troponin and liver enzymes may have been due to ischemia, medication, and direct invasion of HSV into the liver and heart, inducing hepatitis and cardiomyopathy [20].

## Conclusions

This study has implications with regard to improving the diagnostic process and response in rural community hospitals when an immunocompromised patient presents with HSV pneumonia. It is important to keep in mind that HSV pneumonia can cause pulmonary and systemic complications such as myocarditis and hepatitis. When treating infectious diseases, it is important to consider the possibility that immunocompromised patients can experience exacerbations of underlying diseases, requiring systemic treatments.
